# Awareness of Standardised Tobacco Packaging among Adults and Young People during the Final Phase of Policy Implementation in Great Britain

**DOI:** 10.3390/ijerph14080858

**Published:** 2017-07-31

**Authors:** Ilze Bogdanovica, Magdalena Opazo Breton, Tessa Langley, John Britton

**Affiliations:** Division of Epidemiology and Public Health, University of Nottingham, UK Centre for Tobacco and Alcohol Studies, Clinical Sciences Building, City Hospital, Hucknall Road, Nottingham NG5 1PB, UK; Magdalena.Opazo@nottingham.ac.uk (M.O.B.); Tessa.Langley@nottingham.ac.uk (T.L.); j.britton@outlook.com (J.B.)

**Keywords:** standardised packaging, smoking, survey

## Abstract

*Background*: In May 2016, along with the latest European Tobacco Products Directive (TPD), standardised packaging legislation was implemented in the UK. During the following 12-month transition period, both new and old types of packaging were allowed on the market. This study aimed to assess awareness of standardised packaging and other TPD changes in the UK population in March 2017, when both forms of packaging were in widespread use. *Methods*: We conducted two surveys—one in adults and one in young people—investigating awareness of plain packaging legislation. In young people, we also measured susceptibility to smoking using previously validated questions. We asked smokers whether they had recently changed the product they used and also whether they used any of the products that are banned by the new legislation. *Results*: In the adult survey, 73.5% (95% CI: 71.5–75.5%) of the participants were aware of the new legislation and 32.4% (95% CI: 30.3–34.5%) had noticed changes in tobacco packaging, this proportion being considerably higher among smokers (83.7%; 95% CI: 78.9–87.5%) than never smokers (20.7%; 95% CI: 18.2–23.4%). More than half (52.4%; 95% CI: 46.5–58.4%) were using pack sizes or shapes (typically less than 20 cigarettes or 30 g loose tobacco), that would become illegal after full TPD implementation, and 31.4% (95% CI: 26.2–37.1%) reported switching to a different product since October 2016, in most cases to a cheaper brand. Among young people, 20.2% (95% CI: 17.8–22.7%) reported that they had noticed standardised packaging, comprising 16.2% (95% CI: 13.7–19.0%) of non-susceptible never smokers, 25.6% (95% CI: 18.0–35%) of susceptible never smokers, and 49% (95% CI: 37.8–60.2%) of ever smokers. *Conclusions*: In the final stages of implementation, awareness of the introduction of standardised packs was highest among smokers. The TPD will cause nearly half of adult smokers to purchase larger packs, and may cause many smokers to switch to cheaper brands.

## 1. Introduction

Smoking is the largest avoidable cause of death in the United Kingdom (UK), killing around 96 thousand people every year [1]. Although smoking prevalence has reduced over recent years, reaching 15.8% in 2015, there are still 7.6 million smokers in the UK [2]. Preventing smoking therefore remains a high public health priority.

Tobacco packaging has been widely used to market tobacco products, especially so since other marketing channels have been prohibited through tobacco control legislation [3]. Restrictions on pack designs, including prohibition of misleading descriptors and requiring pictorial health warnings have previously been introduced to reduce the appeal of tobacco packaging. On 20 May 2016, the 2014 European Tobacco Products Directive (TPD) [4] and standardised (plain) packaging legislation [5] were implemented in the UK, imposing the most substantial changes to pack designs to date. To comply with the TPD legislation, all cigarettes must be sold in packs containing at least 20 cigarettes and be of a standard shape, while the minimum size for roll-your own (RYO) tobacco pack is 30 g [4]. Restrictions on packaging have also been accompanied by the implementation of larger and updated pictorial health warnings [4]. UK legislation has gone further, with all packs now also required to be in Pantone 448 C colour (drab brown/green) with a matte finish, and branding limited to a brand name and descriptor in standard black font [5].

The legislation allowed a one-year transition period leading to full implementation in May 2017. During the transition year, both branded and standardised packs were allowed to be sold, and during the course of the year standardised packs were gradually introduced. We carried out the survey in the final stages of implementation of TPD and standardised packaging legislation to investigate whether people were aware of plain packaging and had been exposed to it close to the full implementation deadline. The aim of this study was to investigate awareness of standardised packaging legislation among adults and children, when both standardised and branded packs were still widely available. This study will assist policymakers in deciding whether the gradual implementation of the policy is the most appropriate approach and whether the implementation of standardised packaging policy offers opportunities for further smoking reduction interventions.

## 2. Material and Methods

In March 2017, approximately 10 months into the standardised packaging legislation transition period, we commissioned YouGov [6] to carry out online surveys of adults and young people in Great Britain (GB), investigating their awareness of standardised packaging legislation. Authors designed the questionnaire and YouGov was involved in formatting the questionnaire, accessing participants, and collecting the data. The surveys were in field 17–20 March 2017, and were conducted in accordance with the Market Research Society standards and did not require additional ethics approval. Respondents voluntarily sign up to the YouGov panel and a sub-sample of the panel is invited to complete a specific survey. Each respondent was compensated with small financial incentive for their time. For the purpose of respondent verification, demographic questions are regularly updated and sense checked to previous answers, and accuracy of previously given information is checked at the start of the survey if required [7]. Parental approval was given for participation of young people, and consent of a participant was assumed by starting the survey. However, participants could withdraw from the survey at any point and their responses were not included.

Due to the way YouGov collect the data, it was not possible to measure the exact response rate for the adult survey, though the response rate for the young people survey was 27%. However, the completion rate for the children survey was 94%, suggesting that almost all participants who took part in the survey completed it. For the analysis, we used weighted data to be representative in terms of sex, age, social class, and region for Great Britain. Population estimates were presented in the descriptive tables to demonstrate the representativeness of the sample [8,9].

The survey of adults (people aged 18 years and over) elicited information on age, sex, socio-economic status (measured as Approximated Social Grade produced by Office for National Statistics), and region of residence. We asked participants whether they were current smokers, never smokers, or ex-smokers, and then asked them if they were aware of the new plain packaging legislation and whether they had noticed any changes in tobacco packs in the last six months. We categorised all participants into smokers and non-smokers (comprising never smokers and ex-smokers) to investigate noticing changes and being aware of the new legislation. We asked smokers to identify the pack size they use from a list of product sizes currently available on the market—20 or more cigarettes, 17, 18, or 19 cigarettes, 10 or 14 cigarettes, and roll your own in pack sizes of 10 g, 12.5 g, 20 g, 25 g, 30 g, 40 g, 50 g, and other. We then classified these products into two categories: products that will be legal after full legislation implementation (cigarette packs of 20 or more and RYO packs of at least 30 g) and those that will not be available on the market after May 2017. We also asked whether they used any of the products that are affected by the implementation of the new legislation and whether participants had changed the product they usually smoke in the last six months (that is, within the period in which standardised packaging was being implemented).

The survey of young people investigated noticing standardised packaging and susceptibility to smoking among 11–15-year-olds. In addition to information on age, sex, region, and socio-economic status, we asked children whether they had ever smoked, had tried smoking but did not smoke now, or whether they were current smokers. Those that indicated that they were never smokers were asked questions to measure their level of susceptibility to smoking. We used three previously validated questions that have been used in various studies: “Do you think you will try a cigarette soon?” (yes/no); “If one of your friends were to offer you a cigarette, would you smoke it?” (definitely yes/probably yes/probably not/definitely not); and “Do you think you will smoke a cigarette at any time during next year?” (definitely yes/probably yes/probably not/definitely not) [10]. Children who answered “no” to the first question and “definitely not” to the following two were classified as non-susceptible to smoking, and the remainder were classified as susceptible. Those that had tried smoking, were former smokers or experimenters, or current smokers were classified as ever smokers. Children who indicated that they were current smokers were asked about the type of tobacco pack they used, and whether they used any of the packs or products that will be affected by the new legislation, including slim or superslim packs, packs of less than 10 cigarettes, cigarettes with a flavour capsule, or menthol cigarettes (although the ban on menthol as a characterising flavour does not come into force until 2020) [11]. We also asked about the use of electronic cigarettes. Finally, we asked all children whether they had noticed the new plain packaging before taking part in the survey. We were unable to show them a picture of the new packs, as doing so contravened the YouGov ethics guidance.

We used descriptive statistics to analyse data from both surveys, and logistic regression analysis to investigate which characteristics predicted noticing standardised packaging among adult survey participants only as the number of smokers among young people was too low. For the logistic regression, our outcome variable was noticing plain packaging (yes/no), and our explanatory variables included in the model were age, sex, smoking status, social class, and region in Great Britain. All calculations and analyses were conducted by authors using YouGov data. Statistical significance level was set at 0.05 and Stata v.14 (Stata Corp., College Station, TX, USA) was used to conduct the analysis.

## 3. Results

### 3.1. Adult Survey

In the adult survey we obtained data from 2033 participants, of whom 52% were female and more than half were aged over 45 years, with a relatively even distribution across social classes and regions of residence (Table 1), in accordance with Great Britain estimates. After excluding data from 29 participants who did not provide information on smoking status, there were 15.7% (95% CI: 14.1–17.4%) current smokers among our respondents.

All participants were asked whether they had noticed any changes in tobacco packaging in the six months before the survey, and 32.4% (95% CI: 30.3–34.5%) reported that they had. As expected, these changes were noticed more by current (83.7%; 95% CI: 78.9–87.5%) than never (20.7%; 95% CI: 18.2–23.4%) or ex-smokers (25.1%; 95% CI: 21.9–28.6%). Participants had mainly noticed the removal of branding (26.2%; 95% CI: 24.3–28.2%), change in colour (12.8%; 95% CI: 11.4–14.4%), and change in size of the tobacco packs (9.4%; 95% CI: 8.2–10.8%). Respondents were also asked whether they were aware of plain packaging legislation before taking part in the survey, and 73.5% (95% CI: 71.5–75.5%) confirmed that they were. The proportion who had noticed changes was substantially higher for smokers than for non-smokers (Table 2).

After excluding data from the 16 smokers who did not ever smoke the products listed (smoked other tobacco products), more than half of smokers (52.4%; 95% CI: 46.5–58.4%) were using pack sizes or shapes (predominantly cigarette packs containing less than 20 cigarettes, and RYO packs of less than 30 g) that would become illegal after the full implementation of the TPD. There were 20.1% (95% CI: 15.9–25.2%) of smokers who would be affected by the prohibition of menthol cigarettes, and smaller proportions using cigarettes with a flavour capsule (7.6% (95% CI: 5.0–11.4%)) or slim/superslim cigarettes (4.5% (95% CI: 2.7–7.5%)). There were 31.4% of smokers (95% CI: 26.2–37.1%) who reported switching to a different product since October 2016, and most of these (55.9%; 95% CI: 45.1–66.1%) had switched to a cheaper brand, followed by switching to larger packs or e-cigarettes; however, numbers in these categories were small. The percentage of e-cigarette use among current smokers was 21.5% (95% CI: 17.1–26.7%).

In a multivariate logistic regression investigating predictors of awareness of plain packaging, we found that after adjusting for age, region, and socio-economic class, being male and being a smoker were independently related to higher levels of awareness (Table 3). Awareness was also higher in older age groups, but was found to be the lowest in the lowest socio-economic group. The same model also indicated that participants from South West and Wales were significantly more likely to have been aware of plain packaging compared to other regions in Great Britain.

### 3.2. Young People Survey

In the young people survey, we obtained data from 1,041 participants aged 11–15 years, 91.1% of whom (95% CI: 89.2–92.7%) were never smokers. Respondents included representative percentages of girls and boys and were spread evenly across the age range, though they tended to be from families of relatively high socio-economic status (Table 4). From the group of never smokers who answered the questions on susceptibility to smoking, 9.8% (95% CI: 8.0–11.9%) were defined as susceptible to smoking on the basis of their responses.

One in five respondents (20.2%, 95% CI: 17.8–22.7%) reported that they had noticed the new plain packaging, comprising 16.2% (95% CI: 13.7–19.0%) of non-susceptible never smokers, 25.6% (95% CI: 18.0–35.0%) of susceptible never smokers, and 49% (95% CI: 37.8–60.2%) of ever smokers. In the group of current smokers (2.4%; 95% CI: 1.6–3.5%), 48.1% (95% CI: 28.4–68.4%) smoked RYO tobacco, and the remainder smoked cigarettes from a range of different packaging types. Out of all the participants, 1.6% had used e-cigarettes.

## 4. Discussion

### 4.1. Main Findings

This study demonstrates that 10 months into the one-year transitional period for the move to standardised packaging and other TPD legislation in the UK, three in four adults were aware of the new legislation, though when we asked whether they had noticed changes in tobacco packaging six months prior to the survey, only one in three reported that they had. However, only one in five children had noticed the plain packaging. Plain packaging was noticed more by adult smokers than non-smokers, and mainly among children who had ever smoked or were susceptible to smoking. The study also demonstrates that tobacco product pack sizes banned by the TPD are popular and widely used among adults, and that, in particular, the legislation will result in many smokers having to buy larger, and hence more expensive, tobacco packs. A considerable proportion of adult smokers reported having switched to different products, mainly to cheaper brands, larger packaging, or e-cigarettes, in the six months before taking part in the survey.

### 4.2. Strengths and Limitations

To our knowledge, this is the first survey of awareness of standardised packaging implementation in a representative sample of the general population in Great Britain. Although it was not possible to know the exact response rate for adults due to the methods used, and the response rate in the youth survey was low, a comparison of our weighted estimates with national estimates for socio-demographic variables and regional representation suggest that results are broadly representative of the general population in Great Britain. Numbers in some categories of respondents are small, and in particular the number of smokers in the young peoples’ survey was too small to provide reliable insight into product choices. However, our other findings are likely to be generalisable to the wider population. Participants of the YouGov panel received financial incentive for participation in the survey, which might have had implications for participation in the study. However, considering that the amount of financial incentives provided is small (obtained in points that can be collected) and is provided for all surveys that a YouGov panellist takes part in, it is unlikely to substantially affect the results of this study.

### 4.3. Comparison with Previous Research

Research carried out to date indicates that removing branding is likely to reduce the appeal of smoking among young people [12,13,14,15], and this finding was confirmed in a study conducted in Australia after plain packaging was introduced [16]. A recent systematic review of the effects of plain packaging confirmed that tobacco products in plain packs are perceived as less appealing, and that lower quality and plain packaging reduce the misperception that some brands are less harmful than others [17]. This, and the fact that in our study, awareness of standardised packaging was concentrated among smoking adults and susceptible children, suggests that standardised packaging is likely to help to promote both smoking cessation in adults, and discourage smoking initiation in children. The relatively low awareness among non-smokers of the various tobacco packaging changes we studied is likely to reflect the fact that, in the absence of advertising, and with a ban on point-of-sale tobacco product displays now in place across the UK, non-smokers are relatively unlikely to have sight of current cigarette packs.

Our study suggests that during this legislative transition period, smokers have switched to larger packs and different products. Although we cannot attribute this directly to the implementation of standardised packaging legislation and the TPD, our findings are in line with observations from Australia, where the implementation of plain packaging legislation led to many smokers switching to different products and price segments [18]. It is, however, also possible that changes in individual income or expenditure, or price changes unrelated to the implementation of standardised packaging, explain some of the changes in products used, and we did not directly enquire participants about reasons for switching in the questionnaire. Unfortunately, ethical considerations precluded us from showing images of standardised packs with updated health warnings to children, and as we were more interested in measuring awareness of rather than exposure to plain packaging in the adult sample, pictures of the plain packs were not included.

## 5. Conclusions

Overall our findings demonstrate that the phased introduction of standardised tobacco packaging in the UK has not been widely noticed by non-smokers, but that awareness among smokers is high. However, it is apparent that smokers have switched to different products or different sizes of packaging and, considering that some of the products will not be available on the market after the full implementation of the legislation, further switching is likely. This indicates that the full implementation of the new legislation could potentially lead to more switching. In Australia, the implementation of plain packaging was a teachable moment, as it led to increase in the number of quitline calls [19]. Therefore, efforts should be made to encourage smokers to quit or reduce harm rather than to choose an alternative tobacco product in the UK. Also, research investigating switching patterns and strategies used by the tobacco industry to retain people into smoking and provoke young people’s interest in smoking is of crucial importance, and will help to refine further tobacco control policy.

## Figures and Tables

**Table 1:** Descriptive statistics of adult participants.

Variable		Percentage	95% CI	GB Estimates
Sex	Male	48.4	46.2–50.6	49.3
	Female	51.6	49.4–53.8	50.7
Age (years)	18–24	11.6	10.2–13.2	11.2
	25–34	15.1	13.6–16.8	17.2
	35–44	16.1	14.5–17.9	16.1
	45–54	17.7	16.0–19.5	17.9
	55+	39.5	37.4–41.6	37.6
Social Class	AB	28.0	26.1–30.0	22.3
	C1	29.0	27.1–31.0	30.9
	C2	21.0	19.1–23.0	20.9
	DE	22.0	20.2–23.9	25.9
Region	East Midlands	7.3	6.2–8.5	7.4
	East of England	8.4	7.2–9.7	9.6
	London	13.4	11.9–15.1	13.4
	North East	5.1	4.2–6.2	4.2
	North West	10.6	9.3–12.0	11.3
	Scotland	8.7	7.6–10.0	8.8
	South East	14.2	12.3–15.8	14.1
	South West	9.9	8.6–11.3	8.8
	Wales	5.0	4.1–6.0	4.9
	West Midlands	9.1	7.9–10.5	9.0
	Yorkshire and the Humber	8.5	7.2–9.8	8.5

**Table 2:** Noticing changes in packaging by smoking status.

Changes in Packaging	Non-Smokers * % (95% CI)	Smokers * % (95% CI)
Removal of branding	19.3 (17.4–21.3)	62.7 (56.9–68.2)
Changes in colour	6.2 (5.1–7.5)	48.1 (42.3–53.9)
Changes in size	4.2 (3.4–5.4)	36.8 (31.3–42.6)
Noticed any changes	22.5 (20.5–24.6)	83.7 (78.9–87.5)
Aware of plain packaging legislation	70.5 (68.3–72.7)	90.5 (86.4–93.5)

* Smokers include current smokers; non-smokers include never and ex-smokers.

**Table 3:** Determinants of awareness of plain packaging.

Variable		Odds Ratio (95% CI)	*p*-Value
Sex	Female	1.00	
	Male	1.33 (1.07–1.65)	0.010
Age (years)	18–24	1.00	
	25–34	1.17 (0.78–1.76)	0.443
	35–44	1.07 (0.71–1.60)	0.750
	45–54	1.52 (1.02–2.27)	0.040
	55+	1.72 (1.22–2.42)	0.002
Smoking status	Non-smoker	1.00	
	Smoker	4.85 (3.16–7.43)	<0.001
Region	East Midlands	1.00	
	East of England	1.26 (0.76–2.10)	0.370
	London	0.83 (0.52–1.32)	0.432
	North East	0.95 (0.53–1.71)	0.876
	North West	1.36 (0.83–2.21)	0.217
	Scotland	1.28 (0.77–2.12)	0.341
	South East	1.23 (0.77–1.95)	0.381
	South West	1.77 (1.06–2.97)	0.029
	Wales	1.83 (1.00–3.37)	0.049
	West Midlands	0.98 (0.59–1.61)	0.927
	Yorkshire and the Humber	1.53 (0.90–2.60)	0.117
Social class	AB	1.00	
	C1	0.98 (0.74–1.28)	0.869
	C2	0.90 (0.66–1.24)	0.539
	DE	0.64 (0.86–2.32)	0.004

**Table 4:** Descriptive statistics of youth participants.

Variable		Percentage	95% CI	GB Estimates
Sex	Male	51.2	48.1–54.2	51.2
	Female	48.8	45.8–51.9	48.8
Age	11	19.6	17.4–22.0	20.6
	12	19.3	17.0–21.8	20.3
	13	19.8	17.5–22.3	19.7
	14	20.3	17.9–23.0	19.4
	15	21.0	18.5–23.6	20.0
Social Class	ABC1 (highest)	65.6	62.6–68.4	53.2
	C2DE (lowest)	34.4	31.5–37.4	46.8
Region	East	9.7	8.0–11.7	9.8
	London	8.6	7.0–10.5	17.0
	Midlands	18.4	16.1–20.9	13.7
	North	26.1	23.5–28.9	24.0
	Scotland	10.0	8.3–12.0	7.9
	South	21.2	18.8–23.8	22.8
	Wales	6.1	4.8–7.7	4.8
